# Evaluating Permafrost Degradation in the Tuotuo River Basin by MT-InSAR and LSTM Methods

**DOI:** 10.3390/s23031215

**Published:** 2023-01-20

**Authors:** Ping Zhou, Weichao Liu, Xuefei Zhang, Jing Wang

**Affiliations:** 1 School of Geosciences and Resources, China University of Geosciences (Beijing), Beijing 100083, China; 2 Land Satellite Remote Sensing Application Center, Ministry of Natural Resources, Beijing 100048, China; 3 Zhejiang Laboratory, Research Institute of Intelligent Computing, Hangzhou 311121, China

**Keywords:** Qinghai-Tibet Plateau, deformation prediction, LSTM, MT-InSAR, permafrost degradation

## Abstract

Permafrost degradation can significantly affect vegetation, infrastructure, and sustainable development on the Qinghai-Tibet Plateau (QTP). The permafrost on the QTP faces a risk of widespread degradation due to climate change and ecosystem disturbances; thus, monitoring its changes is critical. In this study, we conducted a permafrost surface deformation prediction over the Tuotuo River tributary watershed in the southwestern part of the QTP using the Long Short-Term Memory model (LSTM). The LSTM model was applied to the deformation information derived from a time series of Multi-Temporal Interferometry Synthetic Aperture Radar (MT-InSAR). First, we designed a quadtree segmentation-based Small BAseline Subset (SBAS) to monitor the seasonal permafrost deformation from March 2017 to April 2022. Then, the types of frozen soil were classified using the spatio-temporal deformation information and the temperature at the top of the permafrost. Finally, the time-series deformation trends of different types of permafrost were predicted using the LSTM model. The results showed that the deformation rates in the Tuotuo River Basin ranged between −80 to 60 mm/yr. Permafrost, seasonally frozen ground, and potentially degraded permafrost covered 7572.23, 900.87, and 921.70 km^2^, respectively. The LSTM model achieved high precision for frozen soil deformation prediction at the point scale, with a root mean square error of 4.457 mm and mean absolute error of 3.421 mm. The results demonstrated that deformation monitoring and prediction using MT-InSAR technology integrated with the LSTM model can be used to accurately identify types of permafrost over a large region and quantitatively evaluate its degradation trends.

## 1. Introduction

The permafrost of the Qinghai-Tibet Plateau (QTP) shows a significant and extensive degradation trend as a consequence of global temperature rise, human activities, and massive carbon emissions [1,2]. The active layer in the hinterland of the QTP is increasing and the freezing duration is shortening [3,4]. Some studies estimated that by the year 2100, the global permafrost area might be reduced by 40%, the degraded area will reach 2 × 10^6^ km^2^, and the degradation of permafrost will increase the global temperature by 0.13–0.27 °C [5,6]. Permafrost degradation of the QTP will seriously influence the ecohydrological system, infrastructure stability, and global climate system [7,8]. Thus, monitoring temporal-spatial deformation and predicting the degradation trends of the permafrost can improve our knowledge of the processes regulating the dynamic changes in the permafrost on the QTP.

The Tuotuo River is the source of the Yangtze River, starting from the Jianggudiru Glacier in the south and ending at the foot of Nangi Balong Hill in the east to meet the Dang Qu [9,10]. Permafrost and seasonally frozen ground are distributed in the Tuotuo River Basin. The Tuotuo River Basin is a fragile ecosystem susceptible to external disturbances and promptly responds to temperature increases. In recent years, due to rising global temperatures, glacier ablation, and soil erosion, permafrost degradation in the Tuotuo River Basin has been increasing every year [11,12]. The Tuotuo River Basin and the whole QTP are recognized as one of the world’s most ideal and distinctive ecological and environmental change research areas. Therefore, it is of great scientific significance to strengthen the monitoring of large-scale permafrost deformation and degradation trends in the Tuotuo River Basin.

Synthetic aperture radar (SAR) interferometry (InSAR) is an efficient approach for investigating large-scale displacement of the permafrost. Multi-temporal InSAR (MT-InSAR) techniques, such as persistent scatterer interferometry (PSI) [13], and the Small BAseline Subset (SBAS) method [14], have been developed to monitor spatio-temporal seasonal freeze-thaw deformations and estimate the active layer thickness (ALT) of the permafrost on the QTP [15,16,17,18,19,20,21,22,23,24,25]. In these studies, deformation monitoring of the Qinghai-Tibet Railway, the Qinghai-Tibet Highway, and their surrounding permafrost regions was carried out using multi-source SAR data and various MT-InSAR methods with different spatio-temporal scales. In addition, some studies retrieved ALT from surface displacement measurements detected by MT-InSAR, soil moisture, and soil porosity [17,22].

Tropospheric atmospheric delays due to complex topography, the complexity of the freeze-thaw deformation process, and decorrelation due to seasonal ground snow and vegetation dynamics influence the application of MT-InSAR to permafrost deformation estimations. Several empirical phase-based methods have been proposed to reduce topography-correlated tropospheric-phase delays [26,27,28,29]. The annual deformation signal of the permafrost includes both a long-term deformation trend and periodic seasonal elevation change. The sinusoidal model or the square root of the cumulative degree day of the freeze-thaw model has been commonly used to approximate seasonal oscillations. However, it is difficult to characterize the freeze-thaw process using a simple physical sinusoidal model, and the square root of the cumulative degree day model is generally constructed by a single point temperature, which cannot represent the deformation process on a large-scale [30]. Due to snow melt or vegetation dynamics (e.g., in alpine meadows), strong spatio-temporal decorrelation may occur in an interferometry network. Thus, the New SBAS (NSBAS) or intermittent SBAS method was developed to overcome the lack of critical interferometric linkages [20,23,31].

Permafrost distribution over the QTP was first mapped to evaluate changes in the thermal state using multi-source datasets based on mathematical and physical models [32,33,34] and then with remote sensing and geographic information system (GIS) technology [35,36]. These studies provided good but low-resolution permafrost distribution maps, which required many input parameters and intensive computations. Zou et al. [35] proposed a permafrost distribution map with a 1 km resolution based on the temperature at the top of the permafrost (TTOP) model and remote sensing technology. In the application of InSAR technology to monitor the degradation trends of permafrost, some researchers have employed InSAR-derived time-series deformation to study the degradation trends of permafrost and permafrost distribution. Zhang et al. [19] adopted multi-source SAR and optical remote sensing data to retrieve the deformation and water area of Salt Lake, showing that surrounding frozen soil has expanded at a rate of 20 mm every year since 2016. Lu et al. [37] used Google Earth Engine and InSAR techniques to jointly analyze the Salt Lake expansion event and permafrost degradation on the QTP. They found that lake expansion may accelerate permafrost degradation. Zhang et al. [20] conducted a preliminary study on the regularities of permafrost deformation of the Qinghai-Tibet Railway from Wudaoliang to Tuotuohe, finding a degradation trend in some permafrost. However, they did not study the distribution of permafrost. Wang et al. [38] used the improved NSBAS technique to retrieve the seasonal deformation and line-of-sight (LOS) deformation rate along the whole Qinghai-Tibet Railway and proposed using only InSAR time-series deformations as the basis for classifying the permafrost.

Deformation prediction is essential for monitoring the seasonal permafrost freeze-thaw cycles and its degradation trends. On the other hand, the Sentinel-1 satellite also provides a rich dataset for a time-series displacement prediction task using InSAR. In the past decade, the rapid development of deep learning in remote sensing image processing and analysis has shown promising application potential. Convolutional neural networks (CNN) were used to detect the slow deformation of volcanoes and predict short-term InSAR deformation maps [39,40,41]. Nukala et al. [42] proposed a new method based on recurrent neural networks (RNN), applying Sentinel-1 to predict time-series deformation maps and achieved good prediction performance. LSTM (Long Short-Term Memory) networks address the limitations of gradient explosion and disappearance that older RNN variants may suffer when learning long-term dependencies of data, improving time-series InSAR deformation prediction models [43,44,45]. Wang et al. proposed an innovative InSAR deformation prediction integrated algorithm based on transformer models to accurately predict time-series deformation surrounding Salt Lake [46]. However, the model performed poorly in permafrost regions. In addition, obtaining InSAR time-series deformation datasets is challenging.

This paper used Small BAseline Subset technology to investigate and analyze the seasonal permafrost deformation process and then used InSAR time-series data to classify the permafrost in the Tuotuo River Basin. Finally, the LSTM model was used to predict the deformation of some points in permafrost areas. The results of permafrost classification provide a scientific basis for permafrost distribution mapping in areas with complex topography. Moreover, the deformation prediction results can quantitatively evaluate the degradation trends of permafrost.

## 2. Study Area and Datasets

### 2.1. Study Area

**Figure 1:**
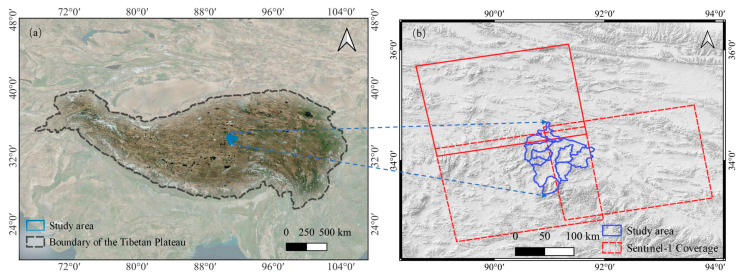
(**a**) Location of the study area with Bing satellite images in the background. (**b**) Sentinel-1A ascending data coverage footprints, shown with Mapzen Global Terrain in the background.

The Tuotuo River Basin is in the southwestern part of the QTP (89°28′48″~92°32′24″ E, 33°13′12″~35°07′12″ N), covering an area of approximately 8490 km^2^ (Figure 1). The average elevation of the basin is over 4600 m. The Tuotuo River Basin is the primary source of the Yangtze River, with the watershed as the western boundary. The south and southeast are bounded by connecting lines of peaks, such as Geladandong, Suojayidoga, Zhabao Shainabao, and Zharigen, and the north is adjacent to the Chumar River basin [9,10]. The geomorphic types of the Tuotuo River Basin are complex, and glacier, permafrost, flowing water, eolian, lake, and swamp geomorphologies are among the main geomorphic types [47]. The climate in the study area is affected by the inland alpine semi-arid and semi-humid climate transition zone, influenced by both moisture flow from the Indian Ocean-Bay of Bengal and westerly disturbances [48]. Meteorologically, temperature, precipitation, and sunshine hours show noticeable spatial-temporal variation [48]. From 1961 to 2018, the annual average temperature and precipitation showed an increasing trend, increasing at a rate of 0.37 °C/10a and 9.86 mm/10a, respectively [47]. Influenced by climate, hydrology, stratum, structure, and permafrost, the occurrence of groundwater and its dynamic distribution characteristics over the study area are spatially heterogeneous [9]. Permafrost plays a critical control role among the influencing factors [9]. The Tuotuo River Basin belongs to an alpine climate, and the permafrost throughout the basin has a depth ranging from 1~3 m. The thickness varies seasonally, and the general annual thawing period is approximately 150 days [49]. In the middle reaches of the basin, influenced by the westerlies, there is a wind transportation-induced accumulation area [50]. Due to the complex landform and zonality of light, heat, and water conditions, the vegetation distribution follows clear horizontal and vertical zoning laws [50].

### 2.2. Datasets

The interferometric wide (IW) swath mode Sentinel-1A ascending data from 14 March 2017 and 6 May 2022, composed of 3 frames covering all of the Tuotuo River Basin, were downloaded from the Alaska Satellite Facility Vertex website [51]. The detailed data information is shown in Table 1. Corresponding Precise Orbit Ephemerides data from Precise Orbit Determination of Sentinel-1 data used for co-registration were downloaded from the Alaska Satellite Facility website [52].

The 1 arc-second resolution SRTM DEM with 30 m ground resolution was used to perform SAR image co-registration, geocoding, and spatial analysis of the deformation results [44]. In this study, the 2 m air temperature, soil temperature, and surface soil moisture ERA-5 reanalysis data were also used to analyze the time-series deformation results of MT-InSAR. The data were acquired from the European Centre for Medium-Range Weather Forecasts (ECMWF) [53].

## 3. Methodology

**Figure 2:**
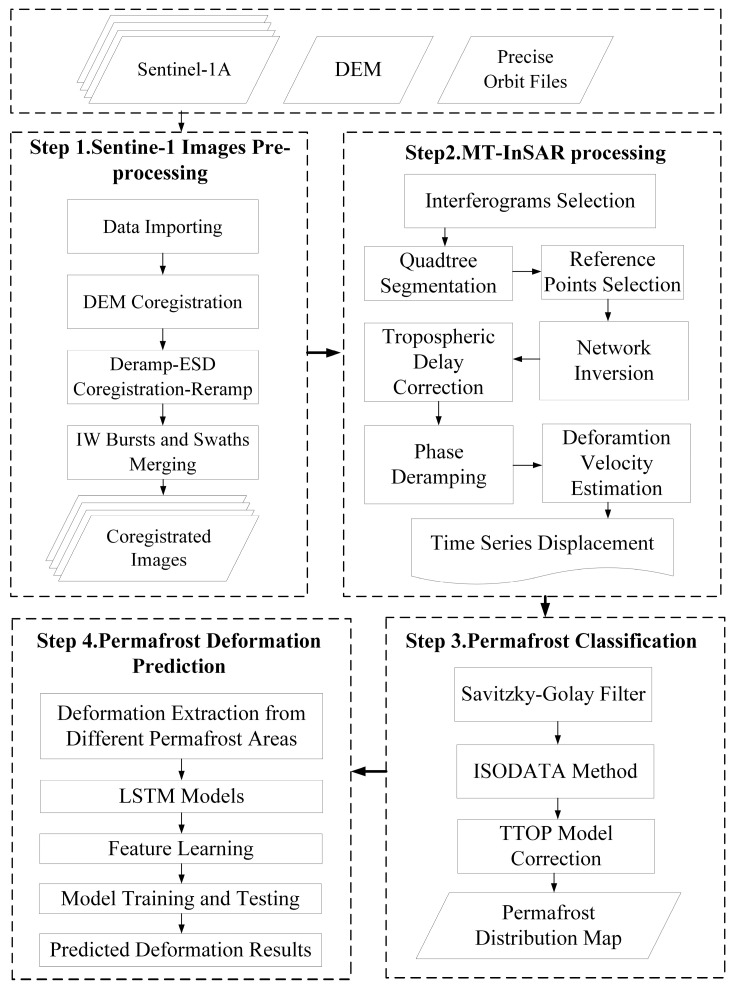
Flowchart of the quadtree segmentation-based SBAS-derived and the LSTM methods.

This paper introduced an LSTM model to predict the surface deformation of different permafrost types in the Tuotuo River Basin. The LSTM model was constructed based on the newly designed quadtree segmentation-based SBAS-derived historical displacement. The four main steps of the process are shown in Figure 2. This flowchart includes the main processing steps: (1) Sentinel-1 image preprocessing. (2) MT-InSAR processing. (3) Permafrost classification. (4) Permafrost deformation prediction.

### 3.1. MT-InSAR Processing

**Figure 3:**
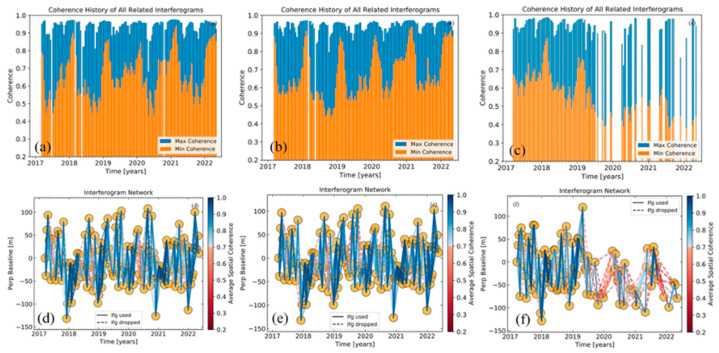
Coherence history of all related interferograms and interferogram networks. (**a**–**c**) The coherence history of all related interferograms in Path 41 Frame 105, Path 41 Frame 110, and Path 143 Frame 106. (**d**–**f**) The interferogram networks of Path 41 Frame 105, Path 41 Frame 110, and Path 143 Frame 106.

We first used the ISCE framework to process Sentinel-1A ascending acquisitions into co-registered interferograms and then filtered and unwrapped these interferograms [54]. To increase the signal-to-noise ratio of each interferogram, all the Sentinel-1 interferograms were multi-looked by factors of 8 and 2 along the range and azimuth directions, respectively. The small baseline network was selected by the spatio-temporal baseline and coherence threshold of the full interferogram image range (Figure 3). We selected interferometric pairs with a 120 m perpendicular baseline and 60 days temporal baseline and coherence of 0.7 as the selection thresholds. To ensure the full connection of the interferogram network, we kept interferograms in the min span tree network based on mean coherence (Figure 3c,f) [55]. Figure 3 shows a high-quality interferogram network in Track 41 (Figure 3d,e). The influence of the low-quality interferograms in the network of Track 143 was reduced by introducing a coherence weight into the deformation inversion process (Figure 3c,f).

A quadtree segmentation-based empirical phase method was designed to reduce the influence of topographic phase noise and correct tropospheric delays in the unwrapped interferograms [56,57]. The advantages of segmentation processing in reducing the impact of atmospheric noise in complex mountain areas have been demonstrated in previous studies [58]. For the segmentation, all interferograms in the modified network were divided into small subsets according to their height difference (threshold of 1000 m by quadtree; [58]). The segmentation process was terminated when the height difference was still greater than 1000 m and the segmented window size reached 10 km across [58]. The segmented interferometric phase was preliminarily corrected in parallel using an empirical phase-based method to reduce the influence of topography-correlated phase noise [55,56,57]. Selecting a stable reference point in each segmentation is essential for deformation inversion from the corrected interferometric phase. However, it is time-consuming to manually select references in all segmented subsets. Thus, an automatic reference point selection strategy for segmented windows was designed by combining the factors of average phase velocity and average spatial coherence [55,58]. In the reference point selection strategy, we first screened stable reference points set, then eliminated the unstable points in the permafrost surface with an average coherence threshold greater than 0.9. Finally, we used the coherence weight-based SBAS method to compute the optimal time series of displacement and deformation velocity of those segmented networks, as implemented in the Mintpy toolbox [55]. The main workflow of the MT-InSAR method includes inversion for the raw phase time series and correcting for phase contributions from different sources to obtain the displacement time series. A detailed description of these two blocks can be found in the study by Yunjun et al. (2019) [55]. The results from each segment were merged after deformation inversion. The results in the overlapping areas were set as the average values from the different segments.

### 3.2. Permafrost Classification

In this paper, an unsupervised classification method, Iterative Self Organizing Data Analysis Techniques Algorithm (ISODATA) [59], based on the InSAR time-series deformation was adopted to accurately classify and map the permafrost distribution map in the Tuotuo River Basin. First, the InSAR time-series deformation of each pixel calculated by the SBAS method was obtained. Second, because the time-series deformation data may have some noise signals, which may be caused by phase unwrapping errors or terrain phase errors, the Savitzky Golay (SG) filtering algorithm [60] was used to preprocess the time-series deformation data. Then, the ISODATA method in ENVI5.3 software was used to implement permafrost classification in the Tuotuo River Basin. The permafrost roof temperature (TTOP) model was introduced to accurately correct the classified permafrost map and allow more accurate classification of frozen soils [35]. The TTOP model is shown in Equation (1):

(1) $$\mathrm{TTOP}=\frac{k_{t}/k_{f}\times TDD-FDD}{P}=\frac{\left(r_{k}\times n_{t}\times I_{t}\right)-\left(n_{f}\times I_{f}\right)}{P}$$

where *P* is the annual period (365 days). *TDD* ($n_{t}\times I_{t}$) is the surface thawing index of permafrost, *FDD* ($n_{f}\times I_{f}$) is the surface freezing index of permafrost, $n_{t}$ and $n_{f}$ denote *n* factors of the thawing and melting seasons, respectively,$\;I_{t}$ and $I_{f}$ are air temperature thawing and freezing index, respectively, and $r_{k}\;\left(k_{t}/k_{f}\right)$ is the thermal conductivity ratio of the soil during freeze-thaw cycles.

Then, the type of permafrost in the study area was further estimated according to the TTOP values using the following equation:

(2) $$D=\{\begin{array}{c} 1,\mathrm{TTOP}\leq 0\;\;\mathrm{permafrost}\;\\ 0,TTOP>0\;\mathrm{seasonally}\;\mathrm{frozen}\;\mathrm{ground} \end{array}$$

Finally, the permafrost distribution map was obtained based on the InSAR deformation information and the TTOP model.

### 3.3. Permafrost Deformation Prediction

**Figure 4:**
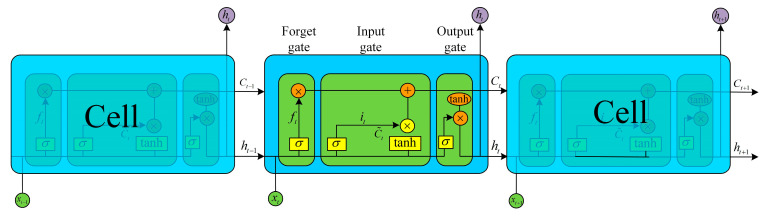
The basic LSTM model structure.

The potential permafrost degradation areas in the Tuotuo River Basin were extracted based on the InSAR deformation information and the permafrost distribution map. LSTM not only solves the long-term dependence problem in Recurrent Neural Network (RNN) [61], but also solves the problems of gradient disappearance and explosion in RNN [62]. The LSTM model (Figure 4) was used to build a surface deformation prediction model and predict the time-series deformation of permafrost in subsequent periods. The memory and gate modules in the LSTM model can learn the deformation characteristics from the time-series data. The gate module contains an input gate, output gate, and forget gate, which can effectively train the fully connected layer to control the cell state in response to the new inputs from the data and past outputs of the model.

The first stage of LSTM determines the forget or remember information in the cell state. This stage is realized by the forget gate. It checks the output at the last moment and the current input, and then determines which information to keep or delete through the sigmoid layer. The equation is as follows:

(3) $$f_{t}=\sigma \left(W_{f}\cdot \left(h_{t-1},x_{t}\right)+b_{f}\right)$$

where σ is the sigmoid activation function,$\;h_{t-1}$ and $x_{t}$ are hidden state and input vector at time t−1, respectively, and $W_{f}$ and $b_{f}$ represent the weight value and deviation value, respectively. If the output value $f_{t}$ is close to 0, the previous data are forgotten. If it is close to 1, it does not mean that the previous data are remembered.

The second stage of LSTM determines which new data will be stored in the cell state. The input gate $i_{t}$ determines which values will be updated. Then, a tanh layer creates a new candidate value vector ${\tilde{C}}_{t}$ which will be added to the cell state. The previous state ${\tilde{C}}_{t-1}$ of the cell is multiplied by $f_{t}$ to express the part that is expected to be forgotten. In the end, the forgotten value is added to the updated state value of the cell $i_{t}*\tilde{C}$, and the current state value of the cell ${\tilde{C}}_{t}$ is obtained as follows:

(4) $$i_{t}=\sigma \left(W_{i}\cdot \left(h_{t-1},x_{t}\right)+b_{i}\right)\;{\tilde{C}}_{t}=\tan h\left(W_{c}\cdot \left(h_{t-1},x_{t}\right)+b_{c}\right)$$

where σ is the sigmoid activation function, $W_{i}$ and $W_{c}$ are the weights of updates, $b_{i}$ is the deviation of the input gate, and $b_{c}$ is the deviation.

In the third stage of LSTM, $f_{t}$ and $i_{t}*{\tilde{C}}_{t}$ are used to update the old cell state $C_{t-1}$, which is multiplied by $f_{t}$ to remove excess information. The new cell state $C_{t}$ is obtained by updating the past state $C_{t-1}$. The calculation is as follows:

(5) $$C_{t}=f_{t}\cdot C_{t-1}+i_{t}\cdot {\tilde{C}}_{t}$$

The final stage of LSTM determines which information to output. First, the output gate determines which parts of the cell state to output, and then the value of the cell state between −1 and 1 is normalized through the tanh function, and the result is multiplied by the output of the sigmoid gate. The output value *h* at the current moment is obtained as:

(6) $$O_{t}=\sigma \left(W_{o}\cdot \left(h_{t-1},x_{t}\right)+b_{o}\right)\;h_{t}=O_{t}tanh\left(C_{t}\right)$$

where $h_{t}\;$ represents the new output value, and $W_{o}$ and $b_{o}$ represent the update weight and deviation of the output gate, respectively.

In summary, a single LSTM neuron can extract features from historical data by the interaction among the forget gate, input gate, output gate, and activation function.

**Figure 5:**
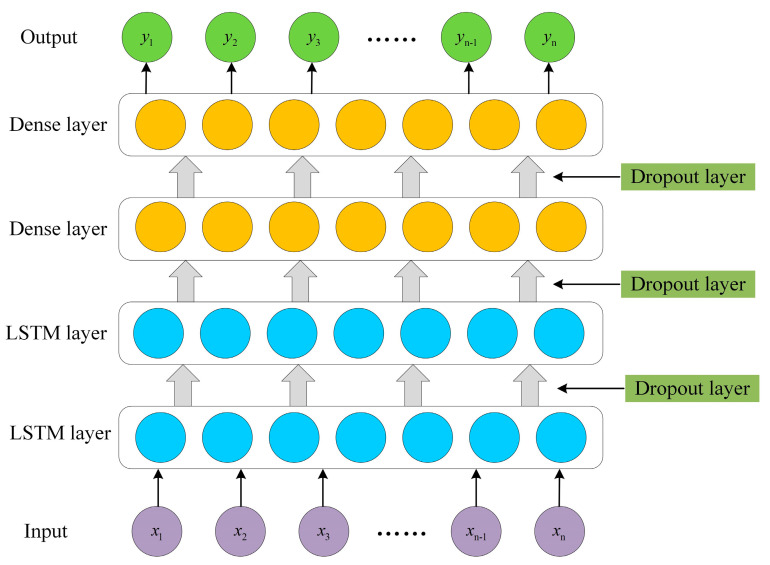
Structure of the LSTM network model for permafrost deformation prediction.

The InSAR monitoring results can be expressed as $x=\left\{x_{1},x_{2},x_{3},\ldots ,x_{n-1},x_{n}\right\}$. The prediction model can effectively learn the change characteristics of time-series deformation data, and the prediction result y of surface deformation can be obtained through the LSTM prediction model, that is, $y=\left\{y_{1},y_{2},y_{3},\ldots ,y_{n-1},y_{n}\right\}$. Figure 5 shows the constructed LSTM model, consisting of a two-layer LSTM layer, two-layer Dense layer, and three-layer Dropout layer.

First, the historical deformations of the measurement points were obtained for different classified permafrost regions, and the LSTM prediction model of ground deformation of the measurement points in the study area was established using the TensorFlow deep learning framework. The InSAR dataset was subdivided to implement the training and testing processes using the LSTM prediction model, with 70% of the time-series deformation data used for training, 20% for testing, and 10% for validation. Second, the min-max normalization method was used to normalize the time-series InSAR deformation data of these deformation measurement points. Then, the network initialization step of the LSTM model was carried out by setting the initialization weight and bias vector, using the Dropout layer to suppress overfitting in the process of network training. In our study, the initial learning rate was 0.0001 and the maximum training time was 2000. The optimizer adopted the Adam (Adaptive Moment Estimation) method. The algorithm calculated the adaptive learning rate under different parameters. The grid search algorithm was used to select the hyperparameters in the prediction model. To evaluate the prediction accuracy of the constructed LSTM neural network model, the Root Mean Square Error (RMSE) and Mean Absolute Error (MAE) were used to evaluate the prediction accuracy of the prediction model.

## 4. Results

### 4.1. LOS Deformation Results

**Figure 6:**
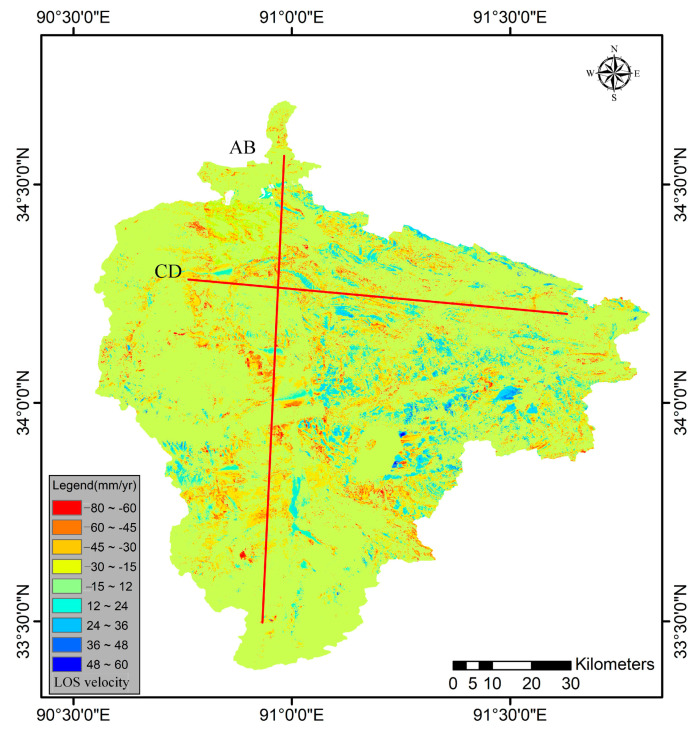
Line-of-sight (LOS) deformation velocities in the study area. And the red line denotes the LOS deformation velocity profile line.

**Figure 7:**
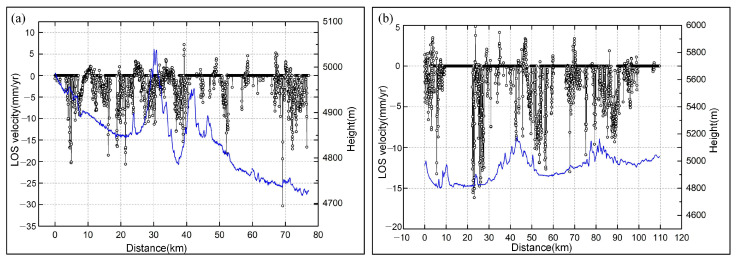
Relationship between elevation (blue) and LOS deformation rate (black). (**a**) LOS deformation velocity profile along A to B. (**b**) LOS deformation velocity profile along C to D.

Based on the designed segmentation-based SBAS method, the LOS deformation velocities of regional permafrost in the study area were obtained from the Sentinel-1A ascending geometries (Figure 6). The LOS deformation velocity ranged from −80 to 60 mm/yr and was generally smaller than 20 mm in most areas, with a mean value of approximately 12 mm/yr. The deformation velocity values of AB and CD profiles were extracted to investigate the spatial distribution characteristics of deformation velocity retrieved from MT-InSAR (Figure 6). A small negative correlation between elevation and deformation velocity was found in both profiles (Figure 7). The reason for this correlation is that the annual average temperature decreases with increasing elevation and the thickness of the active layer is affected by soil properties and decreasing soil moisture, which decrease the deformation trend accordingly. The correlation between topography and seasonal deformation amplitude was also found in the Beiluhe River Basin permafrost region in a previous study [63].

### 4.2. Permafrost Classification

**Figure 8:**
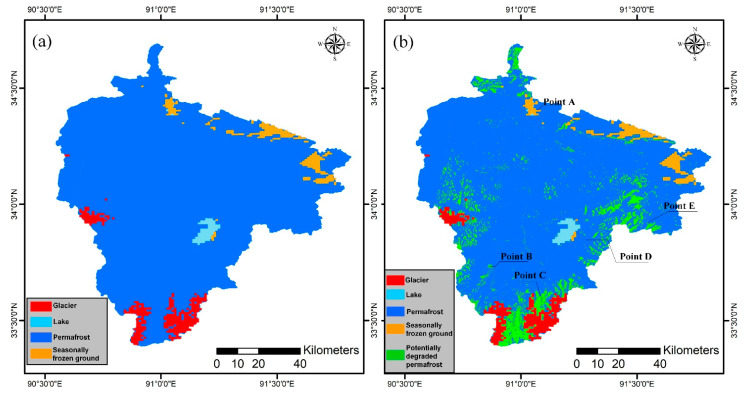
Comparison of frozen soil classification maps between (**a**) ZhaoLin’s method and (**b**) the MT-InSAR method.

Figure 8 shows the classification of frozen soils. Figure 8a shows the classification results based on the TTOP model proposed by Zhao et al. [64]. The results were obtained from the 2017 Tibetan Plateau permafrost classification map of the National Qinghai-Tibet plateau Center [35,64] and clipped using ArcMap 10.6 software. Figure 8b shows the results based on the MT-InSAR time-series deformation using the ISODATA method. The classification results of the two methods are approximately the same, but there is a difference in permafrost areas. The area of seasonally frozen ground obtained by the proposed method was 900.87 km^2^ and the area of permafrost was 7572.23 km^2^.

Due to the degradation of some permafrost into seasonally frozen ground in recent years, the permafrost area has been reduced. In addition, there may be some errors in time-series deformation calculations, such as unwrapping errors, terrain errors, and the influence of soil water content, precipitation, and glacier activity in some areas, which lead to inaccurate time-series deformation calculations in MT-InSAR, resulting in inaccurate classification results [38]. A marked trend of seasonal displacement in permafrost areas was detected using MT-InSAR and the interannual displacement increased yearly. Some permafrost areas were divided into potentially degraded permafrost regions with a significant seasonal deformation characteristic, and the area of potentially degraded permafrost was 921.70 km^2^.

### 4.3. Permafrost Deformation Prediction

**Figure 9:**
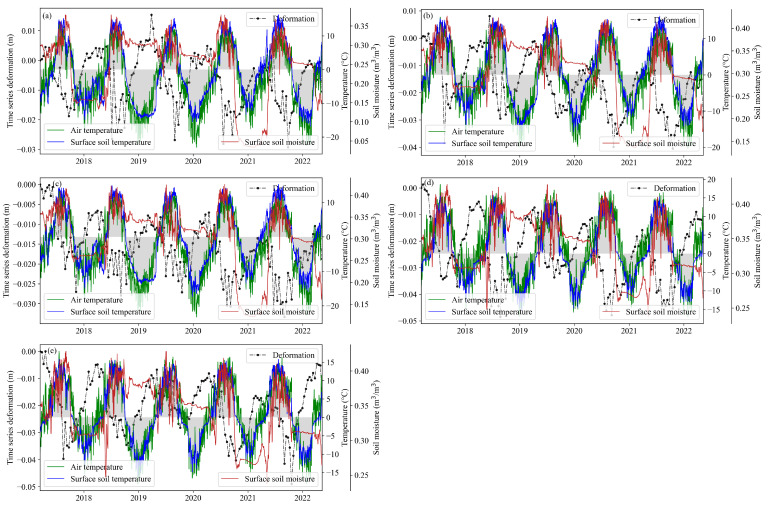
Relationship between the time-series deformation and air temperature, surface soil temperature, and surface soil water content of typical sample points. (**a**) Point A. (**b**) Point B. (**c**) Point C. (**d**) Point D. (**e**) Point E.

The potential degradation areas of permafrost were extracted based on the InSAR information and permafrost distribution map (Figure 9). The time-series deformation of five points in the permafrost areas and potentially degraded permafrost areas were extracted to further analyze the deformation law of different permafrost types. Point A is a deformation point in seasonally frozen ground areas; points B and C are deformation points in potentially degraded permafrost areas; and points D and E are deformation points in permafrost areas.

As shown in Figure 9, a seasonal time-series deformation characteristic was found in the SBAS method used in this study, which demonstrated the effectiveness of the deformation estimation approach used. The time-series deformation was closely correlated with temperature. The key driving effect of temperature on the seasonal deformation of permafrost has been mentioned in previous studies [38,63]. A time lag of temporal deformation to temperature variation may exist for permafrost. Approximately 20 days deformation delays after the air temperature begins to stay above or below 0 °C were found in the Tuotuo River Basin (Figure 8). Figure 9 also displays the positive correlation between soil moisture dynamic and seasonal deformation variation. Thus, apart from temperature, precipitation, soil moisture, and other hydrologic-related factors play a critical role in the temporal deformation of the permafrost.

**Figure 10:**
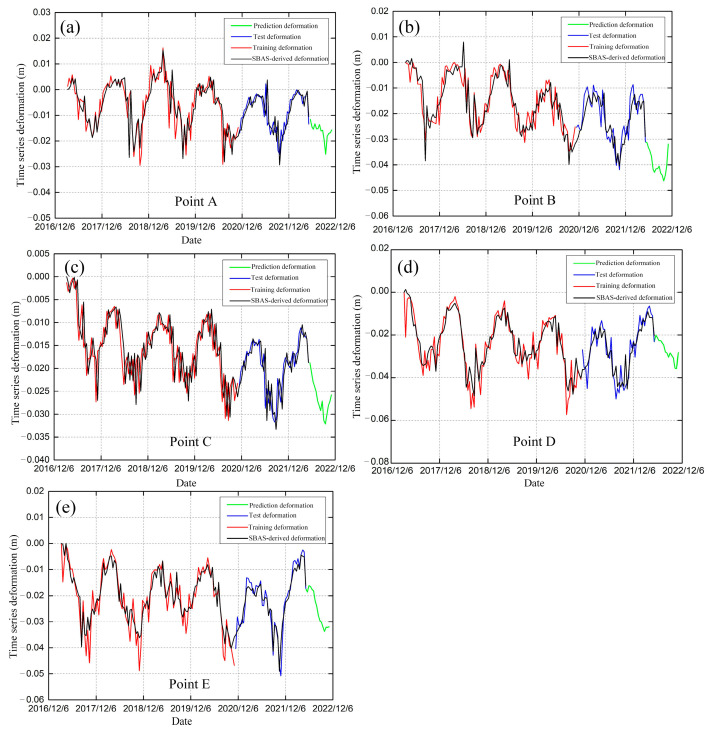
Cumulative deformation predicted by the LSTM model and monitored by MT-InSAR of typical sample points. (**a**) Point A. (**b**) Point B. (**c**) Point C. (**d**) Point D. (**e**) Point E.

**Figure 11:**
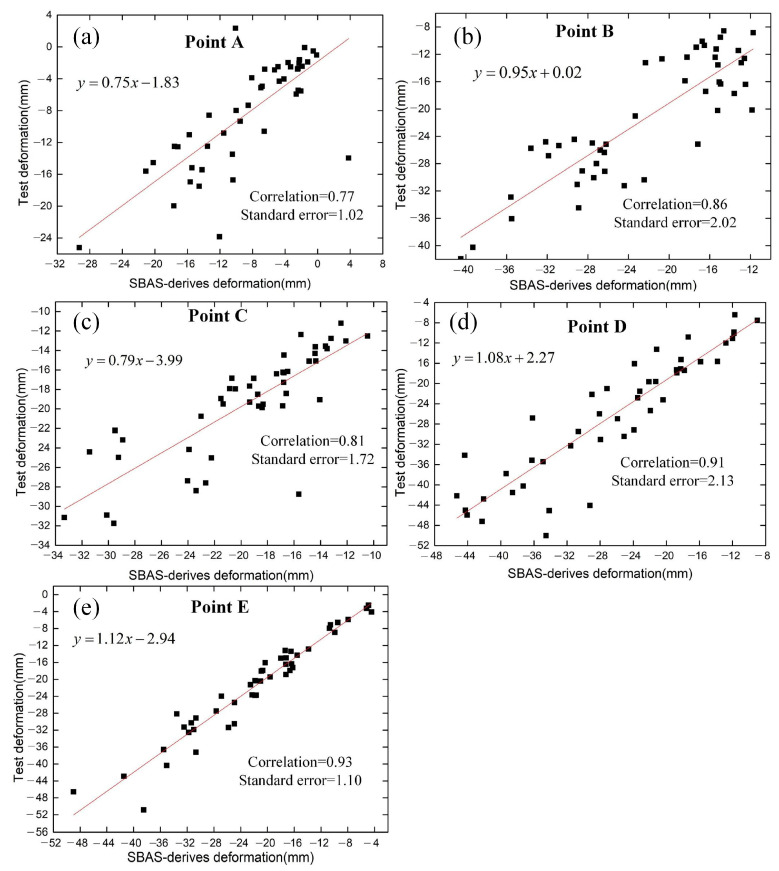
Correlation between SBAS-derived deformation and test deformation of typical sample points. (**a**) Point A. (**b**) Point B. (**c**) Point C. (**d**) Point D. (**e**) Point E.

The LSTM model was used to predict and analyze the time-series deformation of these points. We used 108 historical InSAR time-series deformations as the training dataset, 46 time-series deformations as the test dataset, and 15 time-series deformations as the validation dataset (Figure 10). To ensure the reliability of the prediction results, the LSTM model was used to make short-term predictions for permafrost areas with surface deformations, namely points A, B, C, D, and E in the Tuotuo River Basin. The predicted duration of this experiment was set to the next 6 months. It was found that the prediction results of the LSTM model could predict the freeze-thaw process and periodic deformation changes in permafrost areas. Figure 11 shows the correlation and standard error between the SBAS-derived deformation and test deformation of typical sample points. The monitored deformations of permafrost derived by the SBAS method and the predicted deformations in the test dataset by the LSTM model showed good correlations of about 0.77, 0.86, 0.81, 0.91, and 0.93, respectively. The predicted values were in good agreement with the measured values, indicating that the LSTM prediction model established in this experiment can reliably predict surface deformation.

The prediction results indicated that the temporal deformations of points A, D, and E showed a periodic freeze-thaw process and the predicted results showed a trend of summer melting, which is in line with the law of permafrost deformation, indirectly confirming the accuracy of the prediction results. The maximum cumulative settlement in summer 2022 was expected to be −15.68, −28.18, and −31.96 mm, respectively. Figure 10b,c shows the predicted deformation curves at points B and C, indicating that the permafrost in this region presents a potential degradation trend. The increasing interannual deformation in subsequent months may be related to permafrost degradation. The maximum deformations of the degradation at points B and C were predicted to be −31.88 and −25.80 mm, respectively. To evaluate the accuracy of the predicted time series deformation based on the LSTM model, the root mean square errors (RMSE) and mean absolute errors (MAE) of points A, B, C, D, and E are shown in Table 2. The RMSE values of the training and test datasets at point E were the smallest, at 4.288 and 3.228 mm, respectively. The MAE values of the training dataset at point E and the test dataset at point C were the smallest, at 3.073 and 2.330 mm, respectively. The accuracy evaluation indexes of the LSTM model had better performance in predicting the deformation trend of frozen soils with the seasonal signals.

## 5. Discussion

### 5.1. Comparison with Previous Studies

The challenges of MT-InSAR applications in permafrost areas include temporal decorrelation due to surface dynamics, atmospheric phase screen errors, and seasonal deformation. In this study, the influence of temporal decorrelation was reduced by introducing a coherence weight into the SBAS deformation inversion. The topography-correlated atmospheric phase noise was reduced by quadtree segmentation. Permafrost undergoes seasonal freezing and thawing, which cannot be correctly described by the linear deformation model in the MT-InSAR method. The Stefan model or sinusoidal approximation models are commonly used to describe the seasonal variation processes of permafrost. The applicability of those models has been well demonstrated at the local scale [15]. However, the soil surface temperature of permafrost and seasonally frozen ground show significant spatio-temporal variations. It is unreasonable to characterize the physical processes of different types of permafrost displacement with a single sinusoidal model or the Stefan model in large-scale monitoring. Compared with seasonal deformation model-based MT-InSAR methods, the SBAS method used in this study converted the interferometric phase to a raw phase time series by a weighted least squares estimator. For each pixel, the quality of the inverted raw phase time series could be assessed by minimizing the interferometric phase residual [55]. This process was implemented without relying on seasonal deformation. As shown in Figure 8, the seasonal deformation characteristic of the permafrost was successfully detected, demonstrating the applicability of the non-deformable model-based SBAS in long-term permafrost deformation monitoring.

### 5.2. Advantages of Permafrost Classification and Prediction of Permafrost Deformation

The classification of frozen soils in this paper relied on an unsupervised approach based on the deformation properties of different frozen soil types. In this approach, the classification was based on the time-series deformation of each measurement point and did not need external data as input. There was also no need to build physical and empirical models of frozen soil, which reduced the computational burden. Additionally, the TTOP model was used to accurately correct unclassified permafrost and seasonally frozen areas with the same deformation law, improving the classification accuracy compared to Wang’s results [38]. In the application of InSAR deformation prediction, some researchers have used the deep neural network method to predict the time-series deformation of SAR images, obtaining good prediction performance at the point scale of InSAR time-series displacement [45,46]. For frozen soils areas with seasonal signals, the deformation law is closely related to temperature, soil water content, and precipitation, so the deformation curve presents obvious sine or cosine curve changes. The LSTM model in InSAR deformation prediction has the function of long-term memory, which can better simulate the frozen soil deformation trend. However, it is not easy to parallelize the training process in LSTM models. Due to their periodicity, the length of the sequence modeled by LSTM is limited.

## 6. Conclusions

In this study, we designed a parallel quadtree segmentation-based MT-InSAR method to conduct permafrost deformation monitoring in the Tuotuo River Basin. We classified the permafrost using MT-InSAR-monitored deformation and the TTOP model to predict the degradation trends of different types of permafrost. The deformation characteristics of permafrost were successfully predicted and analyzed based on the LSTM model, and the main conclusions are as follows:

(1) This work demonstrated the applicability of the quadtree segmentation-based MT-InSAR method in monitoring permafrost deformation. It provides an effective strategy for reducing the influence of topography-correlated atmospheric delay by using the empirical phase-based method in the segmented subset. The SBAS method is an effective option for monitoring permafrost deformation without using the permafrost seasonal deformation model. The deformation results showed that the LOS deformation velocity ranged from −80 to 60 mm/yr and was generally smaller than 20 mm in most areas.
(2) Based on the InSAR time-series deformation results and the TTOP model, permafrost in the Tuotuo River Basin was classified as permafrost, seasonally frozen ground, and potentially degraded permafrost. The area of seasonally frozen ground obtained by the proposed method was 900.87 km^2^ and the area of permafrost was 7572.23 km^2^. Additionally, we found that potentially degraded permafrost covered an area of 921.70 km^2^.
(3) The LSTM model successfully predicted the time-series deformation information of different classified permafrost types, showing a trend of interannual deformation subsidence in potentially degraded permafrost areas. The LSTM model can be used to effectively predict InSAR deformation information in complex permafrost areas.

In the future, we will collect additional Sentinel-1 data for permafrost deformation monitoring and prediction tasks, as well as obtain ground measurements to quantitatively assess permafrost degradation trends.

## Figures and Tables

**Table 1:** Sentinel-1 data used in the study area.

Path	Frame	Temporal Span (y/m/d)	Image Counts	Orbit Geometry
41	105	14 March 2017–29 April 2022	140	Ascending
41	110	14 March 2017–29 April 2022	140	Ascending
143	106	21 March 2017–6 May 2022	106	Ascending

**Table 2:** Comparison of accuracy evaluation indexes of the LSTM model during the training and test processes.

Point Index	Training Dataset		Test Dataset	
	MAE	RMSE	MAE	RMSE
Point A	3.489	5.410	2.936	4.550
Point B	3.421	4.457	3.747	4.619
Point C	3.640	4.618	2.330	3.369
Point D	3.727	5.474	3.483	4.994
Point E	3.073	4.288	2.375	3.228

## Data Availability

Sentinel-1 data are provided by the European Space Agency (ESA) and available from the Alaska Satellite Facility (ASF) (https://vertex.daac.asf.alaska.edu). SRTM DEM data are available at https://srtm.csi.cgiar.org/srtmdata/.
